# Tumor Spreading to the Contralateral Ovary in Bilateral Ovarian Carcinoma Is a Late Event in Clonal Evolution

**DOI:** 10.1155/2010/646340

**Published:** 2009-09-15

**Authors:** Francesca Micci, Lisbeth Haugom, Terje Ahlquist, Vera M. Abeler, Claes G. Trope, Ragnhild A. Lothe, Sverre Heim

**Affiliations:** ^1^Department of Medical Genetics, The Norwegian Radium Hospital, Oslo University Hospital, 0310 Oslo, Norway; ^2^Department of Cancer Prevention, Institute for Cancer Research, The Norwegian Radium Hospital, Oslo University Hospital, 0310 Oslo, Norway; ^3^Centre for Cancer Biomedicine, University of Oslo, 0310 Oslo, Norway; ^4^Department of Pathology, The Norwegian Radium Hospital, Oslo University Hospital, 0310 Oslo, Norway; ^5^Department of Gynecology, The Norwegian Radium Hospital, Oslo University Hospital, 0310 Oslo, Norway; ^6^Faculty of Medicine, University of Oslo, 0310 Oslo, Norway

## Abstract

Cancer of the ovary is bilateral in 25%. Cytogenetic analysis could determine whether the disease in bilateral cases is metastatic or two separately occurring primary tumors, but karyotypic information comparing the two cancerous ovaries is limited to a single report with 11 informative cases. We present a series of 32 bilateral ovarian carcinoma cases, analyzed by karyotyping and high-resolution CGH. Our karyotypic findings showed that spreading to the contralateral ovary had occurred in bilateral ovarian cancer cases and that it was a late event in the clonal evolution of the tumors. This was confirmed by the large number of similar changes detected by HR-CGH in the different lesions from the same patient. The chromosomal bands most frequently involved in structural rearrangements were 19p13 (n = 12) and 19q13 (n = 11). The chromosomal bands most frequently gained by both tumorous ovaries were 5p14 (70%), 8q23-24 (65%), 1q23-24 (57%), and 12p12 (48%), whereas the most frequently lost bands were 17p11 (78%), 17p13 (74%), 17p12 (70%), 22q13 (61%), 8p21 and 19q13 (52%), and 8p22-23 (48%). This is the first time that 5p14 is seen gained at such a high frequency in cancer of the ovary; possibly oncogene(s) involved in bilateral ovarian carcinogenesis or tumor progression may reside in this band.

## 1. Introduction

Cancer of the ovary represents 30% of all malignancies of the female genital organs [1]. The most common ovarian neoplasms, typically occurring in women of reproductive age and beyond, originate from the ovarian surface epithelium and belong to one of three major types: serous, mucinous, and endometrioid tumors. These neoplasms range from the clearly benign (80%) to highly malignant carcinomas, with tumors of borderline malignancy in between. 

Bilateral carcinomas of the ovary vary in frequency depending on which tumor type is involved but can be found in roughly 25% of all ovarian cancer cases [2]. The question of whether bilateral ovarian carcinomas are the result of metastatic spreading from one ovary harboring the primary tumor to the contralateral ovary, as opposed to the alternative, simultaneous occurrence of two independent primary tumors, was addressed nearly two decades ago by Pejovic et al. [3]. In a chromosome banding analysis they found no clear-cut difference in the karyotypic pattern between the tumors of the two ovaries in each woman but considerable differences from case to case. Hence, metastatic spreading from one side to the other must have been the pathogenetic mechanism, although the side carrying the primary tumor could not be identified. No similar later studies have been undertaken to confirm or falsify the findings and conclusions then made.

We present a series of 32 bilateral ovarian carcinoma cases, in six also including cancerous lesions in the omentum or peritoneum, analyzed primarily by karyotyping and high-resolution comparative genomic hybridization (HR-CGH) but also tested for microsatellite instability. Because the findings in the two or three samples from each woman were largely similar, we conclude that bilateral ovarian cancer occurs by a metastatic process and that spreading to the contralateral ovary mostly is a late event in the clonal evolution of these cancers.

## 2. Materials and Methods

### 2.1. Tumors

The examined material consists of 70 fresh samples from ovarian carcinomas surgically removed at The Norwegian Radium Hospital from 1999 to 2004 (see Table 1 in Supplementary Material available online at doi:10.1155/2010/646340). The tumors were part of a consecutive series of 248 ovarian tumors cultured and karyotyped by us (of which 203 were carcinomas; unpublished data). The 70 tumors came from altogether 32 patients with bilateral ovarian cancer. From all patients we had tumor material from both ovaries, and from six patients we also had samples from a metastasis to the omentum (three patients) or peritoneal cavity (three patients). Neoadjuvant therapy had been given to four patients before surgery (cases 5, 9, 22, and 24); otherwise, no preoperative chemotherapy or irradiation had been given. The tumors were classified as serous papillary adenocarcinoma (22 cases; Supplementary Table 1), endometrioid carcinoma (cases 23, 29, and 31), adenocarcinoma NOS (case 24), carcinosarcoma (case 21), and mucinous adenocarcinoma (case 26), and four cases showed a mixed histology and were classified as endometrioid and serous papillary (cases 3, 6, and 27) and clear cell and serous papillary carcinoma (case 28). The tumors also showed different patterns of differentiation (well, moderately, and poorly differentiated; Supplementary Table 1).

### 2.2. Cell Culturing and Karyotyping

The tumor samples were manually minced and disaggregated with Collagen II (Worthington, Freehold, NJ, USA) until a suitable suspension of cells and cell clumps was obtained. After 6-7 days of culturing in a selective medium [4], colchicine was added and the cultures harvested according to Mandahl [5]. The chromosomes of the dividing cells were then G-banded and a karyotype established according to the recommendations of the ISCN [6].

### 2.3. High-Resolution Comparative Genomic Hybridization (HR-CGH)

DNA was isolated by the phenol-chloroform method as previously described [7]. CGH [8] was performed according to our modifications of standard procedures [9]. Chromosomes were karyotyped based on their inverted DAPI appearance and the relative hybridization signal intensity was determined along each chromosome. An average of 10–15 metaphases was analyzed. A negative (normal versus normal) and a positive (a cell line with known copy number changes) controls were included in the experiments. For the scoring of CGH results, we adopted the use of dynamic standard reference intervals (D-SRI). A D-SRI represents a “normal” ratio profile that takes into account the amount of variation detected in negative controls for each chromosome band. This provides a more objective and sensitive scoring criterion than fixed thresholds [10–12] and, consequently, a higher resolution. The D-SRI used was generated with data from 10 normal versus normal hybridizations (totalling 110 cells). This interval was automatically scaled onto each sample profile, and aberrations were scored whenever the case profile and the standard reference profile at 99% confidence intervals did not overlap. The description of the CGH copy number changes was based on the recommendation of the ISCN [6].

### 2.4. Microsatellite Instability Status

The tumor's microsatellite instability (MSI) status was determined in all samples using a consensus panel of five microsatellite markers (BAT25, BAT26, D2S123, D5S346, and D17S250) [13]. A tumor was considered to be MSI-high if two or more of the five markers exhibited novel alleles compared to normal DNA, MSI-low if only one marker deviated from the normal pattern, and microsatellite stable (MSS) if none of the tumor genotypes showed an aberrant pattern. Control DNA corresponding to the individual tumors was not available from the patients and therefore single allele changes, that is, the presence of two different alleles, can reflect a heterozygous constitutional genotype or a homozygous genotype with a novel tumor-specific allele. Thus, dinucleotide markers were not scored when such a pattern appeared in the tumors. The MSI status was assessed according to Wu et al. [14]. Allelic sizes were determined using GeneMapper 3.7 software (Applied Biosystems, Foster City, CA, USA), and the results were independently scored by two investigators. A second round of analyses was always performed, confirming the findings.

## 3. Results

**Figure 1:**
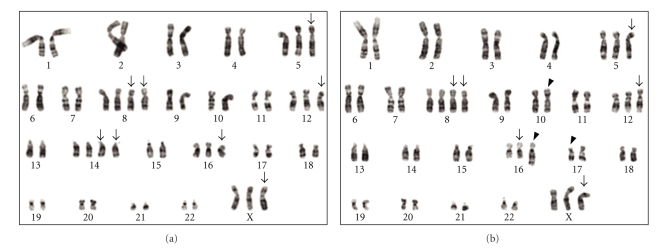
Tumor karyotypes from case 19. The two cancerous ovaries showed an identical karyotype with two related clones: (a) 54,XX,+X,+5,+8,+8,+12,+14,+14,+16, and (b) 52,XX,+X,+5,+8,+8,inv(10)(p12q22),+12,+der(16)t(14;16)(q13;q22),del(17)(p12). Arrows point to numerical changes, arrowheads to structural rearrangements.

The cell culturing and subsequent G-banding cytogenetic analysis gave informative results in 58 samples (Supplementary Table 1), 39 of which showed an abnormal karyotype whereas 19 were normal. The remaining 12 samples were culture failures and therefore could not be examined using this technique. The abnormal karyotypes were complex; that is, more than four abnormalities were present in all informative cases but one, case 21. This case was the only carcinosarcoma of our series; the tumor of the right ovary (case 21b) showed a der(16)t(1;16)(q21;q22) as the sole abnormality whereas a complex karyotype was seen in the tumor of the left ovary (case 21a), but with a similar der(16)t(1;16)(q21;q22) as one of many changes. Mostly many more aberrations than four were seen, several of which could not be completely identified. The modal chromosome number was hypodiploid in six cases, diploid in three, hyperdiploid in three, hypotriploid in one case, hypertriploid in 19, neartetraploid in one, hyperpentaploid in three cases, and a mix of hyperdiploid and hypertetraploid clones in one (case 5a). For 14 cases, we obtained abnormal karyotypes from either both ovaries or from one ovarian tumor and a metastasis in the omentum/peritoneum, but in six of these cases, the technical quality was not sufficiently good to be able to compare the karyotypic data from different lesions in a reliable way. For eight patients, however, it was possible to compare the abnormal karyotypes, and in one additional patient both ovarian tumors and the peritoneal metastasis yielded informative results and could be compared (cases 8a, 8b, and 8c in Supplementary Table 1). In all these nine cases, more or less extensive karyotypic similarities between the samples taken from the different tumor lesions were found, with the number of identical aberrations ranging from one to eight per karyotype. However, because in seven out of these nine cases the karyotypic description was incomplete due to the complexity of the rearrangements, it is possible that more common abnormalities were present. In cases 19a and 19b, the karyotypic description was identical for the two tumorous ovaries (Figure 1). 

**Figure 2:**
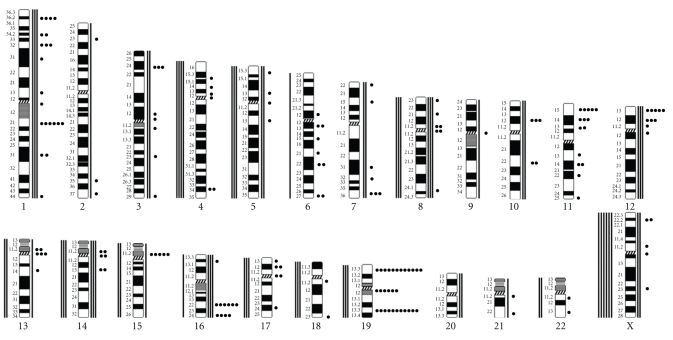
Breakpoint positions (circles to the right) and numerical changes (lines; losses to the left and gains to the right) detected in the chromosome aberrations of 32 cases of bilateral ovarian cancer.

The chromosomes seen to be most frequently involved in structural rearrangements by G-banding analysis were, in order of falling frequency, chromosomes 19, 1, 11, and 16 (Figure 2). The bands most frequently rearranged were 19p13 (involved in 12 rearrangements), 19q13 (11 rearrangements), 1q21, 16q22, and 19q10-11 (six rearrangements), 11p15, 12p13, and 15p11 (five rearrangements each), and 1p36 and 16q24 (four rearrangements each). The chromosomes most frequently involved in numerical aberrations were the X chromosome (in eight tumors) and chromosomes 8 and 14, involved in six tumors each. 

**Figure 3:**
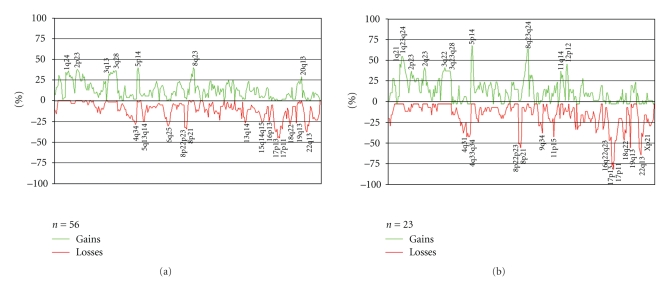
(a) All genomic imbalances detected by HR-CGH in 56 tumor lesions from altogether 32 cases of bilateral ovarian cancer (including six metastases to the omentum/peritoneum). (b) Genomic imbalances detected by HR-CGH in both tumorous ovaries from the 23 cases yielding informative results. The data from the latter subset are likely to reflect the earliest genomic changes, since they are present in both tumor lesions. Some of the imbalances in the former and larger group may have been acquired after spreading, since they also include findings in only one tumor per case.

The HR-CGH gave informative results on 60 samples showing genomic imbalances in 56 of them. From seven lesions there was no DNA available for analysis. No informative results were obtained in cases 13b, 22b, and 32b because of poor quality of the hybridization signal, despite running the experiments twice. In six cases, the G-banding karyotype matched the imbalances detected by CGH well. However, because the G-banding analysis often showed an incomplete karyotype with marker chromosomes and additional material of unknown origin sitting on known chromosomes, the CGH analysis allowed the identification of more imbalances. In 17 cases, a normal karyotype was detected by G-banding analysis whereas the CGH experiments showed genomic imbalances in the tumor samples. We gained information also on the 10 cases that were culture failures, finding imbalances in seven of them. Gains were more frequent than losses as seen by HR-CGH, and high-level amplification was found in 23 lesions. The major copy number changes were gains of or from chromosome arms 1p, 1q, 2p, 3q, 5p, 8q, 11q, 12p, and 20q and losses of or from Xp, 4q, 5q, 6q, 8p, 13q, 16q, 17p, 17q, 18q, 19q, and 22q. More specifically, the most frequently gained bands were, in order of decreasing frequency, 5p14 and 8q23 (39%), 2p23 (38%), 1q24, 3q25, and 3q27q28 (36%), 1q21, and 3q22 (34%), 2p13, 3q13, and 8q21 (32%), 1p31 (30%), 20q13 (29%), and 11q22 and 12p12 (25%). The most frequently lost bands were 17p11 and 17p13 (45%), 17p12 (43%), 16q23 and 22q13 (38%), 8p21 and 17q21 (34%), 8p22-23 (32%), Xp21 and 6q25 (30%), 4q34, and 18q22 (29%), 13q14 and 19q13 (27%), and 5q13-14 (25%) (Figure 3(a)). A comparison of the imbalances scored for the tumors in the two ovaries and/or the omentum/peritoneum showed that the bands most often gained by both ovarian tumors were 5p14 (70% of the 23 cases or 46 samples showing informative results), 8q23-24 (65%), 1q23-24 (57%), 12p12 (48%), 2q23 and 3q22 (43%), and 2p23, 3q13-21, 3q24-28, and 11q14 (39%). The most often lost bands were 17p11 (78%), 17p13 (74%), 17p12 (70%), 22q13 (61%), 8p21 and 19q13 (52%), 8p22-23 (48%), 16q22-23, 17q12-21, and 18q22 (43%), and 4q31, 4q33q34, 11p15, and Xp21 (39% each; Figure 3(b)).

The samples showed from one (samples 4a and 4b) to 58 (sample 11a) copy number alterations with an average number of copy alterations (ANCA) index of 37.9. Amplifications were most often scored on chromosome arms 8q (eight tumors) and 3q and 12p (four tumors each).

As ovarian cancer can be part of the hereditary nonpolyposis colon cancer (HNPCC) spectrum, characterized by microsatellite instability (MSI); we tested for this in the present series. Fifty-nine tumors gave informative results. Fifty-six of the tumors were classified as microsatellite stable (MSS) as none of the tumor genotypes showed an aberrant pattern; these were MSS tumors. Three cases (9a, 9c, and 16b) were scored as MSI-low (MSI-L; Supplementary Table 1). In four cases, the MSI status could not be determined in spite of running the experiments twice. The remaining seven samples were not analyzed as there was no DNA available.

## 4. Discussion

Cytogenetic studies of bilateral ovarian cancer are limited to the one by Pejovic et al. [3] who karyotyped tumors from both ovaries in 15 patients. Because the baseline karyotypes in each tumor pair were identical in the 11 patients from whom informative results were obtained, the conclusion was that the second tumor always arose by spreading of a monoclonal process from the first one. However, since the clonal evolution of the neoplastic cells in the two locations was similar, one could not determine which tumor was primary and which was metastatic. 

We report a series of 32 patients with bilateral ovarian cancer analyzed by karyotyping, HR-CGH, and a microsatellite instability assay. As in the study by Pejovic et al. [3], considerable similarity was observed between the left-sided and right-sided tumors. From one to eight common aberrations were seen by karyotyping in the nine patients from whom informative results for both ovaries/omentum/peritoneum were obtained, averaging 3.22 common aberrations per patient. Because the karyotypic descriptions were incomplete, more common aberrations may have remained hidden among the markers. Indeed, the HR-CGH analysis revealed from one (cases 4 and 31) to 43 (case 1) genomic imbalances common to both or all lesions in the 23 patients from whom informative results for both ovaries/omentum/peritoneum were obtained, giving an average of 25.5 common aberrations per case or patient. Our findings therefore confirm the conclusions of Pejovic et al. [3] that bilateral ovarian cancer occurs via a metastatic mechanism, but the addition of CGH data allowed us to expand on this assessment of the pathogenetic connection between macroscopically discrete tumor lesions: the fact that so many aberrations are common to the tumors in both sides indicates that spreading to the contralateral ovary is a late event in the clonal evolution of the neoplastic parenchyma cells. The aberrations unique to each tumor lesion, on the other hand, in all likelihood arose after the metastasis was set up, in the other-sided ovary or in the omentum/peritoneum. The data are too sparse to conclude with certainty whether the latter metastases differ in any significant way from the tumors situated in the ovaries themselves when it comes to acquired genomic aberrations, but this does not appear to be the case. 

In the present study, the chromosomes most frequently involved in numerical aberrations were the X chromosome and chromosomes 8 and 14. More precisely, the X chromosome was lost in six tumors and gained in two, whereas chromosomes 8 and 14 were involved in numerical aberrations in six tumors each, equally often in gains and losses. These numerical aberrations are well known in ovarian carcinomas [15]. The mechanisms behind their occurrence are unknown as are their pathogenetic effects. 

The chromosome most frequently involved in structural rearrangements was chromosome 19; more specifically, 19p13 was involved in 12 rearrangements in eight tumors, whereas 19q13 was involved in 11 rearrangements in eight tumors, and the centromeric region of chromosome 19 was involved in six rearrangements in three cases. Alterations of 19p13 particularly, but also 19q13, in the form of added extra material are known to be among the most frequent cytogenetic aberrations in ovarian carcinomas [4, 16–18]. Sometimes the 19p+ or 19q+ markers look alike [16, 19], but the origin of the additional material could only rarely be identified [4]. The rearrangements of chromosome 19 have never been seen as the only chromosomal aberration in ovarian carcinoma, and so it seems likely that they are progressional rather than primary anomalies. Their detection here in many cases indicates that they show no frequency-difference between unilateral and bilateral ovarian carcinomas.

Rearrangements of chromosome 1 were also detected quite often in the present series, involving mostly 1q21 (six rearrangements) and 1p36 (four rearrangements). Similar aberrations were also previously reported. Pejovic et al. [16, 19] found frequent deletions of the distal half of 1q and various abnormalities resulting in loss of 1p34-36. Again, no difference between the current series of bilateral carcinomas and ovarian carcinomas in general is discernible. 

Another hot-spot of chromosomal rearrangements in our series was 11p14-15 (eight rearrangements in total, five mapped to 11p15 and three to 11p14). Often the rearrangements are described as an add(11)(p14-15) which, in addition to the added material, may well also lead to loss of 11p-material distal to the breakpoint. Similar changes have been reported by other investigators [16, 20, 21]. Also chromosomal bands 16q22 and 16q24 were repeatedly rearranged in the present series, in six and four cases, respectively. The Mitelman database of chromosome aberrations in cancer reports such changes in five and 14 ovarian carcinomas, respectively [22]. As for the other above-mentioned changes, neither the genes involved nor anything else about the mechanism of their contribution to tumorigenesis is known. 

The karyotypic features of the only bilateral carcinosarcoma analyzed in the present series deserve special mention. The tumor of the left ovary showed two apparently unrelated clones with many abnormalities in an incomplete karyotypic description. The right-sided tumor had a 46,XX,der(16)t(1;16)(q21;q22) as the sole abnormality in all analyzed cells, an aberration that was shared also by the contralateral tumor. In contrast to what appears to be the general rule in the other bilateral tumors, therefore, spreading to the left side seems to have taken place early in clonal evolution in this case. Unfortunately, no DNA was available to perform CGH on the tumor from the right side of this case to compare the genomic imbalances of the two lesions. Carcinosarcomas or malignant mixed mesodermal tumors comprise less than 1% of ovarian neoplasms [23]. They are microscopically characterized by a mixture of malignant epithelial and stromal element, similar to what is observed in corresponding uterine tumors [23]. There are altogether ten such tumors reported with karyotypic aberrations [24–30]. Rearrangement of chromosome 1 seems to be the most frequent cytogenetic change in carcinosarcomas of the ovary as well as of the uterus [9].

The bands most often seen by HR-CGH to be gained in both tumorous ovaries were 5p14 (70% of the 23 cases or 46 samples), 8q23-24 (65%), 1q23-24 (57%), 12p12 (48%), 2q23 and 3q22 (43%), and 2p23, 3q13-21, 3q24-28, and 11q14 (39%). The most often lost bands were 17p11 (78%), 17p13 (74%), 17p12 (70%), 22q13 (61%), 8p21 and 19q13 (52%), 16q22-23, 17q12-21, and 18q22 (43%), and 4q31, 4q33-34, 11p15, and Xp21 (39%). This picture of imbalances by and large tallies well with what has been reported before in unilateral ovarian carcinomas. The most common imbalances detected in ovarian carcinomas by chromosome-based CGH have been gains of or from chromosome arms 1q, 3q, 8q, 12p, and 20q and losses of or from 4p, 4q, 8p, 13q, 16q, 18q, and Xp [31–39]. Interestingly, the chromosomal band most frequently gained in our series of bilateral ovarian carcinomas was 5p14, being gained in 70% of the cases. This is the first time that the short arm of chromosome 5 is seen to be gained so often in ovarian carcinomas. The two only previous reports in which the same band was found gained showed a frequency of 17.6% (three out of 17 sampled analyzed; [40]) and 41.1% (six out of 13 samples analyzed; [41]). In addition, a CGH profile of 543 cases of ovarian carcinomas (summarized in http://www.progenetix.net/) shows a profile for 5p14 with 14.4% gains, 3.9% losses, and 1.3% amplifications. An explanation for the higher frequency of gain of 5p14 observed in our series could be found in the fact that our data are based on high-resolution CGH, a method that is known to be more sensitive in the detection of imbalances compared to normal chromosomal CGH [12]. The most exciting possibility, of course, would be that the observed gain has to do with a tumor's propensity to spread to the contralateral side, but confirmation in independent studies is needed before we ascribe much credibility to this explanation. Regardless of what it might mean, the direct pathogenetic significance of this specific gain remains unknown; possibly oncogene(s) located in 5p14 may be active in ovarian carcinogenesis and/or tumor progression and spreading.

The most frequent losses seen in the present series were from 17p, more precisely 17p11 in 78% of the samples, 17p13 in 74%, and 17p12 in 70%. Much interest has focused on the loss of genetic information from the short arm of chromosome 17, where losses seem to occur especially at 17p13.1 [42, 43] as well as at an even more distal locus in 17p13.3 [42, 44]. Two possible target tumor suppressor genes have been mapped to 17p13.3, *OVCA1* and *OVCA2 *[45]. The target of the more proximal (17p13.1) 17p change could be *TP53.* Indeed, loss at 17p is the most common genetic alteration thus far detected in ovarian cancer, with mutation rates as high as 50% in advanced stage carcinomas [46]. However, we did not perform any further, detailed analysis to see if *TP53* or possibly other gene(s) were directly involved in the losses detected in this series.

DNA microsatellite instability reflects an altered pattern of short tandem repeat sequences (microsatellites) in dividing cells and has been described in HNPCC as well as other tumor types. Ovarian cancer, although most often sporadic, can occur together with HNPCC as part of the Lynch cancer family syndrome [47]. Several studies have suggested an association between MSI and certain histological types of ovarian carcinoma. However, in most of these studies, different kinds of microsatellite markers were used, and the presence of MSI was declared based on the demonstration of instability at only one locus [48–52]. In studies where the NCI markers and criteria were used, Sood et al. [53] found MSI-H in 12% of invasive carcinomas, whereas Gras et al. [54] reported that MSI-H was limited to endometrioid and clear cell carcinomas (12.5%). Liu et al. [55] and Cai et al. [56] found that 20% of endometrioid carcinomas were MSI-H. In our series of bilateral ovarian carcinomas, where 22 of 32 were serous papillary tumors, all cases but one (tumors 9a and 9c; these tumors were serous papillary carcinomas and MSI-L) were MSS. This is in agreement with previous studies finding that most of the MSI were scored in tumors of the clear cell and endometrioid subtype of ovarian cancer.

## Supplementary Material

Supplementary Table 1 is talking about the karyotype, CGH, and MSI information.

## References

[1] Tavassoli FA, Devilee P (2003). Tumors of the breast and female genital organs. *World Health Organization Classification of Tumors*.

[2] Cotran RS, Kumar V, Collins T (1999). *Robbins Pathologic Basis of Disease*.

[3] Pejovic T, Heim S, Mandahl N (1991). Bilateral ovarian carcinoma: cytogenetic evidence of unicentric origin. *International Journal of Cancer*.

[4] Micci F, Weimer J, Haugom L (2009). Reverse painting of microdissected chromosome 19 markers in ovarian carcinoma identifies a complex rearrangement map. *Genes Chromosomes and Cancer*.

[5] Mandahl N, Rooney DE, Czepulkovski BH (1992). Methods in solid tumors. *Human Cytogenetics—A Practical Approach, vol II, Malignancy and Acquired Abnormalities*.

[6] ISCN (2005). *An International System for Human Cytogenetic Nomenclature*.

[7] Brandal P, Bjerkehagen B, Heim S (2004). Molecular cytogenetic characterization of tenosynovial giant cell tumors. *Neoplasia*.

[8] Kallioniemi A, Kallioniemi O-P, Sudar D (1992). Comparative genomic hybridization for molecular cytogenetic analysis of solid tumors. *Science*.

[9] Micci F, Teixeira MR, Haugom L, Kristensen G, Abeler VH, Heim S (2004). Genomic aberrations in carcinomas of the uterine corpus. *Genes Chromosomes and Cancer*.

[10] Kirchhoff M, Gerdes T, Rose H, Maahr J, Ottesen AM, Lundsteen C (1998). Detection of chromosomal gains and losses in comparative genomic hybridization analysis based on standard reference intervals. *Cytometry*.

[11] Kirchhoff M, Gerdes T, Maahr J (1999). Deletions below 10 megabasepairs are detected in comparative genomic hybridization by standard reference intervals. *Genes Chromosomes and Cancer*.

[12] Ribeiro FR, Diep CB, Jeronimo C (2006). Statistical dissection of genetic pathways involved in prostate carcinogenesis. *Genes Chromosomes and Cancer*.

[13] Boland CR, Thibodeau SN, Hamilton SR (1998). A national cancer institute workshop on microsatellite instability for cancer detection and familial predisposition: development of international criteria for the determination of microsatellite instability in colorectal cancer. *Cancer Research*.

[14] Wu Q, Lothe RA, Ahlquist T (2007). DNA methylation profiling of ovarian carcinomas and their in vitro models identifies HOXA9, HOXB5, SCGB3A1, and CRABP1 as novel targets. *Molecular Cancer*.

[15] Micci F, Heim S, Heim S, Mitelman F (2009). Tumors of the female genital organs. *Cancer Cytogenetics*.

[16] Pejovic T, Heim S, Mandahl N (1992). Chromosome aberrations in 35 primary ovarian carcinomas. *Genes Chromosomes and Cancer*.

[17] Jenkins RB, Bartelt D, Stalboerger P (1993). Cytogenetic studies of epithelial ovarian carcinoma. *Cancer Genetics and Cytogenetics*.

[18] Thompson FH, Emerson J, Alberts D (1994). Clonal chromosome abnormalities in 54 cases of ovarian carcinoma. *Cancer Genetics and Cytogenetics*.

[19] Pejovic T, Heim S, Mandahl N (1989). Consistent occurrence of a 19p+ marker chromosome and loss of 11p material in ovarian seropapillary cystadenocarcinomas. *Genes Chromosomes and Cancer*.

[20] Panani AD, Roussos C (2006). Non-random structural chromosomal changes in ovarian cancer: i(5p) a novel recurrent abnormality. *Cancer Letters*.

[21] Panani AD (2007). Preferential involvement of chromosome 11 as add(11)(p15) in ovarian cancer: is it a common cytogenetic abnormality in cancer?. *Cancer Letters*.

[22] Mitelman F, Johansson B, Mertens F (2009). Mitelman database of chromosome aberrations in cancer. http://cgap.nci.nih.gov/Chromosomes/Mitelman.

[23] Kurman RJ (2002). *Blaustein's Pathology and of the Female Genital Tract*.

[24] Atkin NB, Baker MC (1979). Chromosome 1 in 26 carcinomas of the cervix uteri. Structural and numerical changes. *Cancer*.

[25] Atkin NB, Pickthall VJ (1977). Chromosomes 1 in 14 ovarian cancers. Heterochromatin variants and structural changes. *Human Genetics*.

[26] Fletcher JA, Kozakewich HP, Hoffer FA (1991). Diagnostic relevance of clonal cytogenetic aberrations in malignant soft-tissue tumors. *The New England Journal of Medicine*.

[27] Guan X-Y, Cargile CB, Anzick SL (1995). Chromosome microdissection identifies cryptic sites of DNA sequence amplification in human ovarian carcinoma. *Cancer Research*.

[28] Pejovic T, Heim S, Orndal C (1990). Simple numerical chromosome aberrations in well-differentiated malignant epithelial tumors. *Cancer Genetics and Cytogenetics*.

[29] Pejovic T, Alm P, Iosif SC, Mitelman F, Heim S (1996). Cytogenetic findings in four malignant mixed mesodermal tumors of the ovary. *Cancer Genetics and Cytogenetics*.

[30] Tanaka K, Boice CR, Testa JR (1989). Chromosome aberrations in nine patients with ovarian cancer. *Cancer Genetics and Cytogenetics*.

[31] Iwabuchi H, Sakamoto M, Sakunaga H (1995). Genetic analysis of benign, low-grade, and high-grade ovarian tumors. *Cancer Research*.

[32] Arnold N, Hagele L, Walz L (1996). Overrepresentation of 3q and 8q material and loss of 18q material are recurrent findings in advanced human ovarian cancer. *Genes Chromosomes and Cancer*.

[33] Sonoda G, Palazzo J, du Manoir S (1997). Comparative genomic hybridization detects frequent overrepresentation of chromosomal material from 3q26, 8q24, and 20q 13 in human ovarian carcinomas. *Genes Chromosomes and Cancer*.

[34] Tapper J, Sarantaus L, Vahteristo P (1998). Genetic changes in inherited and sporadic ovarian carcinomas by comparative genomic hybridization: extensive similarity except for a difference at chromosome 2q24-q32. *Cancer Research*.

[35] Suzuki S, Moore DH, Ginzinger DG (2000). An approach to analysis of large-scale correlations between genome changes and clinical endpoints in ovarian cancer. *Cancer Research*.

[36] Kiechle M, Jacobsen A, Schwarz-Boeger U, Hedderich J, Pfisterer J, Arnold N (2001). Comparative genomic hybridization detects genetic imbalances in primary ovarian carcinomas as correlated with grade of differentiation. *Cancer*.

[37] Sham JST, Tang TC-M, Fang Y (2002). Recurrent chromosome alterations in primary ovarian carcinoma in Chinese women. *Cancer Genetics and Cytogenetics*.

[38] Israeli O, Gotlieb WH, Friedman E (2003). Familial vs sporadic ovarian tumors: characteristic genomic alterations analyzed by CGH. *Gynecologic Oncology*.

[39] Partheen K, Levan K, Osterberg L, Helou K, Horvath G (2004). Analysis of cytogenetic alterations in stage III serous ovarian adenocarcinoma reveals a heterogeneous group regarding survival, surgical outcome, and substage. *Genes Chromosomes and Cancer*.

[40] Helou K, Padilla-Nash H, Wangsa D (2006). Comparative genome hybridization reveals specific genomic imbalances during the genesis from benign through borderline to malignant ovarian tumors. *Cancer Genetics and Cytogenetics*.

[41] Blegen H, Einhorn N, Sjovall K (2000). Prognostic significance of cell cycle proteins and genomic instability in borderline, early and advanced stage ovarian carcinomas. *International Journal of Gynecological Cancer*.

[42] Godwin AK, Vanderveer L, Schultz DC (1994). A common region of deletion on chromosome 17q in both sporadic and familial epithelial ovarian tumors distal to BRCA1. *American Journal of Human Genetics*.

[43] Saretzki G, Hoffmann U, Rohlke P (1997). Identification of allelic losses in benign, borderline, and invasive epithelial ovarian tumors and correlation with clinical outcome. *Cancer*.

[44] Phillips NJ, Ziegler MR, Deaven LL (1996). A cDNA from the ovarian cancer critical region of deletion on chromosome 17p13.3. *Cancer Letters*.

[45] Schultz DC, Vanderveer L, Berman DB, Hamilton TC, Wong AJ, Godwin AK (1996). Identification of two candidate tumor suppressor genes on chromosome 17p13.3. *Cancer Research*.

[46] Schuijer M, Berns EMJJ (2003). TP53 and ovarian cancer. *Human Mutation*.

[47] Arzimanoglou II, Lallas T, Osborne M, Barber H, Gilbert F (1996). Microsatellite instability differences between familial and sporadic ovarian cancers. *Carcinogenesis*.

[48] Fujita M, Enomoto T, Yoshino K (1995). Microsatellite instability and alterations in the hMSH2 gene in human ovarian cancer. *International Journal of Cancer*.

[49] Ohwada M, Suzuki M, Saga Y, Sato I (2000). DNA replication errors are frequent in mucinous cystadenocarcinoma of the ovary. *Cancer Genetics and Cytogenetics*.

[50] King BL, Carcangiu M-L, Carter D (1995). Microsatellite instability in ovarian neoplasms. *British Journal of Cancer*.

[51] Tangir J, Loughridge NS, Berkowitz RS (1996). Frequent microsatellite instability in epithelial borderline ovarian tumors. *Cancer Research*.

[52] Haas CJ, Diebold J, Hirschmann A, Rohrbach H, Schmid S, Lohrs U (1999). Microsatellite analysis in serous tumors of the ovary. *International Journal of Gynecological Pathology*.

[53] Sood AK, Holmes R, Hendrix MJC, Buller RE (2001). Application of the National Cancer Institute international criteria for determination of microsatellite instability in ovarian cancer. *Cancer Research*.

[54] Gras E, Catasus L, Argelles R (2001). Microsatellite instability, MLH-1 promoter hypermethylation, and frameshift mutations at coding mononucleotide repeat microsatellites in ovarian tumors. *Cancer*.

[55] Liu J, Albarracin CT, Chang K-H (2004). Microsatellite instability and expression of hMLH1 and hMSH2 proteins in ovarian endometrioid cancer. *Modern Pathology*.

[56] Cai KQ, Albarracin C, Rosen D (2004). Microsatellite instability and alteration of the expression of hMLH1 and hMSH2 in ovarian clear cell carcinoma. *Human Pathology*.

