# Supplementary material for: Tumor Spreading to the Contralateral Ovary in Bilateral Ovarian Carcinoma Is a Late Event in Clonal Evolution
Source: J Oncol. 2009 Sep 15;2010:646340. doi: 10.1155/2010/646340 (PMC2744120; doi:10.1155/2010/646340)
Supplement: Supplementary file 1 — Supplementary Table 1 is talking about the karyotype, CGH, and MSI information. [file 646340.f1.pdf]

Table 1. Bilateral Ovarian Carcinomas Examined by Karyotyping, High Resolution-CGH, and Microsatellite Instability Analysis (karyotypic similarities are indicated in bold)

Case/lab number	Diagnosis*	Karyotype	HR-CGH imbalances	MS-status **
1a/49-99	serous papillary cystadenocarcinoma, stage 3c, left ovary	46,XX[14]	rev ish enh(1p13,1p32p36,1q21q24,1q41q42,2p11,2p13, 2p21,2p22p24,2q14,2q36,3p14p21,3p23,3p25,3q13q22,5p, 5q11,5q12q13,5q33q34,8p11p12,8q23q24,9p12p21,9q13q21, 9q32q34,10p11,10p13p14,10q11q21,10q22,10q24q26, 11p11p12,11p14p15,11q13,11q22,11q23q25,12p12p13, 12q23q24,13q32,15q24q26,19p13,20p11p13,20q),dim(X,4,6, 8p21pter,13q12q21,15q14q21,16q21qter,17,18,19q13, 22q11q13)	ND
1b/49-99	right ovary	71~77<3n>,XX,-X,+1,+3,-4, -6,+7,-8,+9, +10,-13,-17,-18, add(19)(p13)x2,+21, +3~9mar[cp11]	rev ish enh(1p11p21,1p22,1p32pter,1q21q31,1q32q43, 1q44,2p11,2p13,2p21,2p22p25,2q11q12,2q14,2q35q37, 3p14p21,3p22p25,3q21q23,5p,5q11q13,5q32q34,7q11,7q22, 7q32q36,8p11p12,8q12q21,8q22qter,9p12p24,9q13q22, 9q32q34,10p11p15,10q11,10q21q26,11p11p12,11p14p15, 11q12q13,11q22q24,12p12p13,12q22q24,13q31q32, 15q24q26,19p13,20p11p13,20q,21q21,21q22),dim(X,4,6, 8p21pter,13q12q21,15q14q22,16q13qter,17,18,19q13, 22q11q13)	MSS
2a/54-99	serous papillary adenocarcinoma – poorly differentiated, stage 4, left ovary	46,XX[27]	rev ish enh(1p31p34,1q,2p11p25,2q11q12,3q12q13,3q24q28, 5p12p15,6p,7q,8p11,10p14p15,11p11,12p,12q12q21,14q, 16p11,16q12q13,18p11,19p13,19q13,20p11p13,20q,21q22), dim(Xp21pter,Xq21q28,3p14p26,4p13pter,5q14,6q16qter, 7p21pter, 8p12pter,9p13p24,13q13qter,16q22q24,22q), amp(12p12pter,12q13q14)	MSS

2b/55-99	right ovary	56~81,add(11)(p13),add(14)(p11),add(18)(q23)x2, add(19)(q13),inc[cp8]/ 46,XX[8]	rev ish enh(1p32p34,1q21q32,2p11p25,3q25q28,5p,6p11p21, 6p22p25,7q,8p11,8q21,10p15,11p11,12p,12q12q21, 16q12q21,18p11,19p13,20p11,20q13),dim(Xp21p22, Xq26qter,3p22p26,4p13pter,6q22,6q24q27,8p12pter, 9p21p24,13q14q34,16q23qter,22q12qter)	MSS
2c/56-99	Omentum	80~85,inc[2]/46,XX[101]	rev ish enh(1p32p34,1q31,1q32q41,2p11p14,2p15p25, 3q25q29,5p13p15,6p22pter,7q31q35,8p11,8q12q21,8q23, 10p11,12p12pter,12q12q21,15q25qter,18p11,20q11q13), dim(3p14p21, 6q21q22,6q24q25,9p21p23,10q24,15q23q24, 16p13,16q21q24,17p13,17q21,22q11qter)	MSS
3a/63-99	endometrioid and serous papillary adenocarcinoma - poorly differentiated, stage 4, right ovary	culture failure	no imbalances	MSS
3b/64-99	left ovary	culture failure	no imbalances	MSS
4a/65-99	serous papillary adenocarcinoma- moderately differentiated, stage 2c, right ovary	culture failure	rev ish enh(1q21q43)	MSS
4b/66-99	left ovary	46,XX[86]	rev ish enh(1q)	MSS
5a/00-23***	serous papillary adenocarcinoma – poorly differentiated, stage 3c, left ovary	49~95,del(6)(q12),inc[2]/ 46,XX[3]	rev ish dim(1p36,7q11,9q34,11q13,12q24,17p11p13, 17q11q25,19)	ND

5b/00-24	right ovary	46,XX[46]	rev ish enh(8q24),dim(7q11,17p11,19p13,19q13)	MSS
6a/00-71	endometrioid and serous papillary adenocarcinoma, stage 3c, right ovary	70~89,add(3)(p12),add(3)(p24),add(6)(q12),add(7)(p22),add(12)(p12),**add(16)(q24)**,inc[3]/ 139~158,idemx2[4]	rev ish enh(1p31pter,1q21q25,1q32q41,2p16p23,2q33q34, 2q35qter,3p14p21,3q13qter,4p16,5p13pter,6q13q14, 7p11p14,7q11q31,7q32qter,8q23qter,9q13q22,10p11, 10p12p14,11q12qter,12q24,14q13q31,15q23q25,16p11p12, 19,20,22q11),dim(Xp11p22,Xq13qter,3p24pter,4q21qter, 5q13q23,6q16qter,8p12pter,10q25qter,11p,13q13q31, 13q32q34,15q11q21,17p,18q21qter,21)	MSS
6b/00-72	left ovary	51~80,add(6)(q27)x2, **add(16)(q24)**x2,inc[2]/ 46,XX[10]	rev ish enh(1q21q43,2q22q32,5p13pter,5q13,5q14, 8q23qter,10p13p15,10q11q21,12p11p13,17q21q25), dim(Xq22q25,1p33p35,3p13p24,4q22qter,6q24qter,8p21pter, 8q13q21,9p21p23,11p12pter,11q22q24,13q21qter,14q13, 14q23q24,16q21qter,17p,17q11q12,18q21q23,19q13, 22q11qter)	MSS
6c/00-73	peritoneum	46,XX[12]	rev ish enh(1p13p31,1p36,1q21q24,1q32q41,2p22p24,2q14, 2q22q37,3q21qter,4p15p16,5p14p15,5q31qter,6p12p23, 6q13q15,7q21qter,8q12,8q24,9p21p23,9q33,10p11p13, 10q22,11q13q14,12p11pter,14q12q21,14q22q31,14q32, 15q24,20p11p13),dim(Xp21,Xp22,Xq21q27,4q24qter, 5q14q23,6q21q22,8p21p23,10q25qter,11p11p14,11q22q25, 12q21q24,13q14q34,15q11q15,16p13,16q13q23,17p,17q21, 18p11,18q21qter,21q,22q13)	MSS
7a/00-118	serous (papillary) cystadenocarcinoma – poorly differentiated, stage 3c, right ovary	54~58,add(7)(q32),add(8)(p11),add(10)(p12),add(14)(p11),inc[cp5]/ 90~113,idemx2[cp6]	rev ish enh(1p13p31,1q21,1q31q43,2p13pter,2q21q37, 3p12p14,3q,5p13p15,6p11p21,6p22,6q12q16,7q21q22, 8q21qter,9p21pter,10q21q22,11p11p14,11q13q23,12p, 12q13q22,13q21qter,14q12q21,14q22q31,15q25q26, 20p11p12,20q11q13),dim(Xp11p21,Xq21,Xq23qter, 1p34p35,4q24qter,5q14q15,5q33q34,6q26q27,7p11p21, 7q34qter,8p12pter,9q13q22,10p11,10p13pter,13q12,	MSS

			15q11q14,15q15q21,16p13,16q21qter,17p11p13,17q11q21,17q22q23,19p13,21q,22q11qter),amp(3p24)	
7b/00-119	left ovary	46,XX[38]	rev ish enh(1p13p31,1q21q31,1q41q42,2p12pter,2q11q23,3p12p21,3q,5q23q33,6p11p21,6q12q14,7q11q33,8q21qter,9p13pter,10q11q22,10q25,11p11p12,11q14q23,12p,12q14q21,13q21qter,20q),dim(Xp,Xq13,Xq21qter,3p24p25,4p13p14,4q13q22,4q25q26,4q31qter,5p13p14,5q12q21,6q21q27,8p21pter,9q22,10p13p14,12q24,13q12q14,14q21q24,14q31q32,15q13q24,16p12pter,17p11p13,17q11q22,18p11,18q12qter,19p13,22q11q13),amp(9p23)	MSS
7c/00-120	peritoneum	46,XX[99]	no DNA available	--
8a/00-206	serous papillary cystadenocarcinoma – mostly moderately and poorly differentiated, right ovary	116~162,**add(4)(p15)**,**add(5)(p13)**,del(7)(q31),der(8)t(8;13)(p23;q14),i(9)(q10),**add(11)(p15)**x2,**add(15)(p11)**x2,inc[cp6]/46,XX[94]	rev ish enh(Xq21,1p13p34,1q21q32,2p,2q11q12,3p12p14,3q,5p13pter,6p23,7p21,7q21,7q31q32,8p11p12,8q,9p12,9q21q33,10p13p14,10q25,11p11p14,12p11p12,12q12q21,14q24qter,20q12qter,21q),dim(Xp11pter,2q35q37,5q11q15,5q23q32,5q34qter,6q24q26,8p21,10q11q22,11p15,11q12q13,15q11q25,16,17,19p13,19q13,22q)	MSS
8b/00-207	left adnex	102~125,-1,**add(4)(p14)**,**add(5)(p15)**,**add(11)(p14)**,add(13)(p11),**add(15)(p11)**,add(16)(p13),inc[cp4]/46,XX[68]	rev ish enh(Xq13,Xq21q26,1p13p21,1p22p36,1q21q25,1q31q32,2p11p25,2q11,2q13q14,2q21,3p13p21,3q13qter,5p13pter,6p21p25,7p12p14,7q11,7q21,7q22q31,8q11q21,8q24,10q25q26,11p12p14,12p12,12q13,14q24qter,19p13,19q13,20q11qter,21q21qter),dim(4q13,4q28,4q34,9p23p24,13q22,16q)	MSS
8c/00-208	peritoneum	53~68,i(5)(p10),**add(11)(p14)**,add(11)(q25),inc[cp12] / 46,XX[79]	rev ish enh(1p31p34,1q23q24,2p12pter,3p12p13,3q12q28,8q23,9q13q22,10q24q25,20q13),dim(Xq11qter,5q11q14,8p21p22,16p11p13,16q13q24,17p11p13,17q11q23,19q13,22q11qter)	MSS
9a/ 00-253***	serous papillary adenocarcinoma, stage 4,	71~84,del(1)(p32)x2, add(11)(p14),inc[cp5]	rev ish enh(Xq12,Xq13q21,Xq23q24,1p31,3q12q13,4p12p13,4q12q13,4q26q31,8q21qter,11q13q24,13q22q31,15q13q14),dim(1p34,4q22q23,5q13,5q34qter,6q21qter,	MSI-L

	right ovary		7p21pter,8p23,9p13,9q21qter,10q23qter,11p15,12q12q15,17p11p13,17q11q22,18p11,18q,19q13,21q22,22q11q13)	
9b/00-254 ***	left ovary	113~116,inc[5]	no DNA available	--
9c/00-255 ***	omentum	75~76<3n>,X,-X,add(X)(p22),add(6)(q21),add(17)(q24),add(19)(p13),inc[cp6]/46,XX[5]/92,XXXX[5]	rev ish enh(Xq21,1p21p22,1q23q25,2q22q24,2q31q32,3p12p14,4p12p13,4p14,4q12q21,4q26q31,5p14p15,6q12q13,8q12qter,10q21,11q13qter,14q11q12,14q21,14q24q32,20q13),dim(Xp11p21,1p34pter,4q22,5q21q22,5q35,6q21qter,7p11p13,7p15pter,7q11q31,7q31q33,8p22p23,9p12p21,9q21qter,10q23qter,11p15,12q12q21,13q12,13q14,16p11p13,16q22q24,17p,17q11q21,18p,18q11,18q12q23,19,21q22,22q11q13)	MSI-L
10a/00-487	serous papillary adenocarcinoma–poorly differentiated, stage 4, left adnex	33~40,XX,add(1)(p34),inc[8]	rev ish enh(1p22,1p31,1q24,1q42qter,2p22p24,3p12p14,3p22p24,3q12q13,3q22qter,4p15pter,4q13,5p14,7q22q31,8q12q24,9q13q22,9q22q33,11q13q22,11q23q25,13q31q32),dim(Xp11p22,Xq13,Xq21q23,Xq24q26,2q14q21,2q34qter,4q23q26,4q31,4q34qter,5q13q14,5q23q31,6q23q26,7p21p22,8p12p23,9p13p23,10q11q21,10q25qter,11p15,13q12q21,14q22qter,15q12q14,16p11p13,16q13q24,17p,17q11q21,18p11,18q21q22,19p13,19q,20p13,21q22,22q11q13)	MSS
10b/00-488	right adnex	38~42,XX,add(1)(p36),add(4)(q34),add(6)(q22),add(11)(p15),add(12)(p13),add(15)(p11),inc[cp6]/65~78,XXX,idem,add(6)(q22),add(12)(p13),inc[cp14]/ 46,XX[6]	rev ish enh(2q23q24,2q32,3q21qter,4p15,4q13,5p14,5q32q33,6p22pter,6q14q21,8q13qter,13q32),dim(Xp21p22,Xq21,Xq24,Xq25q26,2q14,2q37,3p21p24,4q35,5q13q14,6q23qter,7p22,8p12p23,10q21q22,10q25qter,11p15,12p12p13,12q24,13q12q21,14q31qter,15q12qter,16p12p13,16q22q24,17p11p13,17q11q21,18q21q23,19q13,20q12,21q22,22q11q13)	MSS
11a/00-694 ****	serous papillary adenocarcinoma – poorly differentiated, stage 4,	76~86<3n>,XX,-X,+1,del(3)(q23),-4,-5,del(6)(q15),**add(7)(q36)**,**-8**,+10,add(12)(p13),i(13)(q10),**-14,-15**,	rev ish enh(1p13p21,1p22p32,1q21q32,1q32q42,2p11,2p13,2p21pter,2q22q23,3p25p26,3q13,3q21qter,4p15,5p,6p11p21,6p22,6p24,6q12q16,6q22q23,6q26q27,7p12p13,7q11qter,8q13,8q22q24,10p11,10p13p15,10q11q21,10q22q23,	MSS

	left ovary	**add(16)(q24)**,der(19)t(8;19)(q24;q13),**+20**,der(22)t(19;22)(p13;q11),inc[cp9]	10q24q26,11q22,11q24,12p11,12q12q13,18q11q22,19p13,19q13,20,21q21),dim(Xp,Xq12q21,Xq21q27,2q37,5q23qter,7p22,8p11pter,9q22qter,11q12q13,12q14qter,13q12q21,13q21,14q11q31,15q11,15q13q24,15q26,16p12pter,16q,17,18p,19p13,22q)	
11b/00-695 ****	right ovary	67~80<3n>,XXX,+add(1)(p36),**add(7)(q36)**,**-8**,add(11)(p13),-13,**-14**,**-15**,add(15)(p11),**add(16)(q24)**,-17,?i(17)(q10),-18,der(19)(19pter→19q13::hsr19p13→19q11:),der(19)(19pter→19q11::19p13→19p13::19p13→19q11:),der(19)t(3;19)(q12;q13)**+20**,-22,inc[cp5]	no DNA available	--
12a/00-953	serous papillary adenocarcinoma – poorly differentiated, stage 3c, right ovary	46,XX[16]	rev ish enh(1p13p31,1q21q31,2p,3p12,3p12p26,3q12q13,3q22q28,4q12q25,4q26q35,5p13pter,6q14q21,6q22q23,7p13p21,7q21,7q22q36,8q21,10p,11p12p15,11q14q22,13q,15q,16p12pter,20p11p13),dim(Xp22,1q32qter,2q13q21,2q24q31,2q33qter,4p15pter,5q11q14,5q23qter,6p21pter,8q24,9p12p13,9q34,10q21qter,11q13,12p,12q12q14,12q22qter,14q11q24,17p,18p11,18q12q23,19p,19q13,22q),amp(15q26)	MSS
12b/00-954	left ovary	46,XX[4]	rev ish enh(1p21p31,1q23q31,2p,3p12p14,3p22p23,3p25,4q13,5p14p15,6q15q16,7p14p21,7q31,8q22,8q23,10p,11p15,11q14q22,13q21q33,15q,20p11p13,21q21),dim(Xp11,1p33p35,1p36,1q32qter,4p14,5q11q14,5q23q33,7q11,9p12p13,9q22qter,10q21qter,11q12q13,12p11p13,12q12q14,12q15,12q21qter,14q11,14q12q24,14q31q32,16q21q23,17p,	MSS

			17q21,18p11,18q21q22,19,22q)	
13a/00-955	serous papillary adenocarcinoma – poorly differentiated, stage 3c, right ovary	culture failure	rev ish enh(Xp22,Xq13q21,Xq23q25,Xq26q28,1p21p31, 1q31q42,2p,2q11q34,3p12p21,3q,4p12p13,4p15p16, 4q12q21,4q28q31,5p13pter,5q14,5q34,6p12p21,6q12, 8p22pter,8q,9p13pter,9q13q22,10p13pter,11p13p14,12q14, 14q24q32,16q23qter,18q11q21,18q22q23,20q13), dim(1p33p36,3p24p25,4q22q27,4q32qter,5q13,6q25qter,7p, 7q11,8p12,9q33q34,10q11q24,11p15,11q14q24,12p12p13, 12q21qter,13q,14q12q23,15q,16p11p13,17p,17q11q24, 19q13,20p11,20p13,22q12q13),amp(2p15p21,2p24pter, 3q24q29,8q12q21,8q23q24,10p15)	MSS
13b/00-957	left ovary	culture failure	not informative	MSS
14a/00-977	serous papillary cystadenocarcinoma, stage 3c, right ovary	culture failure	rev ish enh(1q21q22,1q41q42,2p11p12,2q11q22,3p12,3q, 4q12q13,5p13,5q31q32,6p11p21,6p21p23,6q12q16,6q16q21, 7p12,8q23q24,9p12p23,12p,12q12q13,13q21qter,14q11q21, 16p11,19p13,19q13,20p11p13,20q11q12),dim(Xp21p22, 4p15p16,4q26q34,5q12q21,8p12p22,8p23,10q11q21,11p15, 11q22,11q23q24,13q12q14,14q24qter,15q14q21,15q22q24, 17p11p12,17p13,18q22q23)	MSS
14b/00-978	left ovary	54~69,inc[2]	rev ish enh(2p11p13,2q11q24,2q31q32,3p12,3q,4q12q21, 4q22q26,5p13p15,6p11p21,6p22p23,6q12q16,8q12q21, 8q22q24,9p12p24,10p11p14,12p,12q12q13,13q21qter, 14q11q21,18p11,18q11q21,19p13,19q13,20p11p13), dim(Xp21p22,3p14p21,3p23p24,4p16,4q31qter,5q12q15, 7q11,7q22,7q34q36,8p11p23,9q22qter,10q11q21,10q23q25, 11p15,11q12q14,11q23q25,12q24,13q12q14,14q23qter, 15q12q24,16q13q24,17p11p13,17q11q21,18q22)	MSS
15a/00-1001****	serous papillary cystadenocarcinoma –poorly	90~99,add(X)(p22),add(1)(p36),del(1)(q31),add(13)(p11),add(17)(p13),der(19)	no DNA available	MSS

	differentiated,<br>stage 3c,<br>left ovary	(19pter→19q11::21q11→21q22::2p23→2pter),+r,inc[8]		
15b/00-1002	right ovary	65~86,add(1)(p34),add(7)(p15),inc[5]/ 46,XX[21]	rev ish enh(Xq23qter,2p11p25,3p12p14,3p21p24,3q12q28,7p11p12,7p13p21,7q11q33,8p11p12,8q,10p11p15,10q11q21,14q11),dim(Xp11pter,1p33pter,2q37,4q33qter,5q12q21,5q23q31,6p12pter,6q25,8p21p23,9p23p24,9q34,14q31qter,16q21qter,17,18p11,18q21qter,21q21q22)	MSS
16a/00-1087	serous papillary adenocarcinoma – poorly differentiated,<br>stage 3c,<br>right ovary	46,XX[14]	rev ish enh(1p22,1p31,1q21q31,2p21,2p22p24,2q14,2q22q23,2q24,2q31q32,3p12p14,3p22p23,3q12q24,3q25,3q27qter,4p15,4q13,5p13pter,5q14q23,6p21pter,6q15q16,8q21qter,9p12p23,9q21,10q11q21,10q25qter,11q14qter,13q31q32,14q21,18q12,21q21),dim(2p11p13,2q33qter,4q31qter,6q22qter,7p21pter,7q21q31,7q31qter,8p11p21,10p12p13,10q22q24,11p15,12p,12q12q14,12q24,13q14q22,15q,16p11p13,16q21q24,17p11p13,17q24q25,20p11p13,20q12,22q13), amp(5q15q21,8q22qter)	MSS
16b/00-1088	left ovary	49~54,+r,inc[3] /46,XX[2]	rev ish enh(Xp21,Xp22,Xq12q27,1p13p31,1q21,1q41q43,2p11p25,2q11q14,2q23q24,5p13pter,5q14q22,5q32qter,6p21,6q21q23,7p11p21,7q11q21,8q12q13,8q21qter,9p13p23,10q,11p11p14,11q13q23,12q15q23,13q12,13q22q34,14q11q21,14q24q32,15q22qter,18q11qter,19q13,20q12q13,21q,22q11q12),dim(1p33p36,2q33qter,3p12pter,3q12q28,4p12p14,4q12q34,5q12q13,6p22,6q12q16,7q21q22,8p12p23,9q34,10p12p15,11p15,12p11p13,12q12q13,12q24,13q14q21,15q11q21,16p12p13,16q21q24,17p11p13,22q12),amp(8q23q24,10q26)	MSI-L
17a/01-805	serous papillary adenocarcinoma – moderately and	67~92,XXX,del(1)(q31),**add(12)(p12)**,inc[3]/ 46,XX[4]	rev ish enh(1p13,1p21p22,1p31,1q21q31,1q32q44,2p11p13,2p16,2p21,2p23p24,2q11,2q13q14,2q21q24,2q31q36,3q,4p15p16,5p13p15,5q11q23,5q32q33,6p12p21,6p22,6q12q15,	MSS

	poorly differentiated, stage 3c, right ovary		8p11p23,8q12q23,10p11,10p13p15,10q24q25,11q13q22, 11q24,12p,13q21,13q22q32,15q24q26,20p11p13,20q), dim(X,1p34,3p14p21,4q,6q16qter,9p12p23,9q21qter, 10q11q22,11p11p15,12q13,12q14,12q15q21,12q23q24,16p, 16q13qter,17p,17q11q22,17q23,18,19q13,21q21qter,22q), amp(12p)	
17b/01-806 ****	left ovary	68~88<3n>,XX,-X,+1,+3, +7,der(7)t(5;7)(q13;q36)x2, der(8)t(8;19)(p11;q13)x2, **add(12)(p12)**x2,+13, der(13;14)(q10;q10)x2,+14, +15,-16,-17,-18,-19, add(19)(q13),der(19) (19pter→19q13::12p11→ 12p13::Xp11→Xpter),-22, +2~16mar[cp10]	rev ish enh(1p21p31,1q23q25,2p23p24,2q14,2q21q24,2q31, 2q32,2q35,3p12p21,3p21p26,3q12q13,3q21q24,5p13, 5p14p15,5q11q23,5q32q34,6q14q16,6q22,7p14p21,7q31, 7q34,8q,9p21p24,11p12p15,11q14q22,11q23q24,12p, 13q21q32,15q24q26,20p12),dim(X,1p32p35,4,7q11, 8p12p23,9q22q31,10p11p14,10q,12q23q24,14q23q24, 14q31q32,16p11p13,16q13q24,17,18,19p13,19q,21q, 22q11q13),amp(12p)	MSS
18a/01-837	serous papillary adenocarcinoma – poorly differentiated, stage 3c, left ovary	Culture failure	rev ish enh(Xq,1p13,1p21p31,1q,2p11p21,2q11q36,3p12p14, 3q12q28,4p14p15,5p12p15,5q14q35,6p,6q12q24,7q21, 7q32q36,9p,9q13q21,10p11,10p13p15,10q11q21,11q13qter, 12p,12q12,13q21q33,14q11q21,16q12,18q11q12,20p11p13, 21q21),dim(Xp11p22,1p32pter,2p22pter,3p21,4q12q28, 4q28q35,5q12q14,7p11p13,7q11,7q22q31,8p11p23,8q12q22, 8q24,9q31qter,10q22q24,11p11pter,12q15q21,12q23q24, 13q12q14,14q22qter,15q11q24,16p12,16q21qter,17p, 17q11q21,18q12q23,19p13,19q13,21q22,22q),amp(3p12p13, 3q12q13,3q22q26)	MSS
18b/01-838 ****	right ovary	70~79,XX,+i(X)(p10),i(X) (p10),dup(1)(q21q44),add(2) (q37),add(3)(q29)x2,i(4) (q10),add(6)(q27),add(11) (q14),i(17)(q10),der(19)	rev ish enh(Xq12q13,1p12p13,1q21q43,2p11p21,2q11q32, 2q33q36,3p12p14,3q12q28,4p15p16,5p13p15,5q21qter, 6p11p24,6q12q16,6q22q23,7q32q36,9p12p23,9q13q21, 10q11q21,10q25,11q12q25,12p,12q12,12q23q24,14q11q12, 16p11p13,17q21qter,19p13,19q13,20p11p13,20q11q12),	MSS

		t(6;19)(p12;q13),der(19)(19pter→19q11::19q11→19p13::18q11→18qter),inc[cp12]	dim(Xp11p22,2p24pter,4q13qter,5q12q14,7q31,8p12p23,8q13q22,9q31q32,10q22q23,11p14p15,12q14q21,13q12q13,14q21qter,15q14q22,16q22q23,17p,18q12q23)	
19a/02-192	serous papillary adenocarcinoma – well differentiated, stage 3b	**54,XX,+X,+5,+8,+8,+12,+14,+14,+16**[1]/**52,XX,+X,+5,+8,+8,inv(10)(p12q22),+12,+der(16)t(14;16)(q13;q22),del(17)(p12)**[11]	rev ish enh(5,8,12,14q,16p,16q12q21,16q22q23),dim(21q21)	MSS
19b/02-193		**54,XX,+X,+5,+8,+8,+12,+14,+14,+16**[5]/**52,XX,+X,+5,+8,+8,inv(10)(p12q22),+12,+der(16)t(14;16)(q13;q22),del(17)(p12)**[9]/46,XX[3]	rev ish enh(Xp22,Xq21q23,Xq24,Xq25,Xq27q28,1p35pter,5p12p14,5p15,5q12qter,8,12,14q,16p,16q12q21,16q23q24),dim(13q31)	MSS
20a/02-706	serous papillary adenocarcinoma – poorly differentiated, stage 3c, left ovary	46,XX[20]	rev ish enh(1p21p31,1q22qter,2p16,2p23,2q14q32,2q33q35,3q,5p,7p12p21,7q21q31,8q12qter,12p12,12q21,18q12,21q21q22),dim(Xp,Xq12q27,1p34p36,3p21,4p16,6p21,6p22,8p12pter,9p21,9q22,9q33qter,11q13,17p11,17p13,17q11,19p13,19q13,22q12q13)	MSS
20b/02-707	right ovary	50~55,X,-X,+der(1)(1qter→1p36::1q21→1qter),+2,+add(8)(p21),+12,+16,-19,-22,+3~8mar[cp6]	rev ish enh(1p13p34,1q21qter,2p12pter,2q11q37,3q,5p,7p12p21,7q21q36,8q,10p11p14,10q24,10q25q26,12p11p13,12q12q13,12q13q24,16q21,18q12q21,19p13,19q13,20q11q13,21q21q22),dim(X,1p36,5q34qter,6p21p22,8p12pter,9p21p23,9q13q21,9q33qter,11q12q14,14q23q24,14q31q32,15q14,15q15, 15q21,15q22q25,17p11p13,19p13,19q13,22q11qter)	--
21a/02-1149	carcinosarcoma (heterologous type), stage 4,	46,XX,add(16)(q22)[4]/66,del(1)(p32)x2,+del(1)	rev ish enh(1q22qter,3p,3q12q13,3q24qter,5p,5q11,5q14q31,5q31q34,6p22pter,6q16q22,7q31,8q,11p11p15,11q13q24,12p12,13q21q32,14q,18p,18q11q21,18q21q22,20),dim(X,	MSS

	left ovary	(q11)x2,+der(2)t(2;12)(q35;q11)x2,add(16)(q22), **der(16)t(1;16)(q21;q22)**, inc[cp4]	1p32pter,2q37,3q21q22,4,6q22qter,9p12p13,9q22qter, 12q22qter,15q,17p,17q21q23,19p13,19q13,22q13), amp(3q25q27,6q21,20p11pter)	
21b/02-1150	right ovary	46,XX, **der(16)t(1;16)(q21;q22)**[11]	no DNA available	--
22a/03-246***	serous papillary cystadenocarcinoma, stage 3c, right ovary	culture failure	no imbalances	MSS
22b/03-247***	left ovary	46,XX[5]	not informative	MSS
23a/03-375	endometrioid adenocarcinoma – well differentiated, stage 1c, right ovary	culture failure	rev ish enh(1q,2q22q24,3p14,3p22p23,5q14,5q21,9p23, 13q22),dim(1p31p36,5q35,7q11,9q22,9q33qter,11q13, 12q23q24,15q22q23,16p11p13,17p11p13,17q11q21, 17q22q25,19p13,19q,22q11q13),amp(1q24q31,1q32)	MSS
23b/03-376	left ovary	culture failure	rev ish enh(1p22,1q,2q22q24,3p12p13,3p23,5q14,5q15, 5q21,6q22,8q22,9p13p21,9p23,13q22,13q31),dim(Xp11, Xq12,1p32pter,2p16,7p11p12,7q11q21,9q22,9q33qter, 10p12,10q23,11q12q13,12p13,12q23qter,13q12,15q22q24, 16p11p13,16q21q24,17p,17q11q21,17q22q25,19p13,19q13, 20q11q12,22q11q13),amp(1q24q31)	MSS
24a/03-518***	adenocarcinoma NOS– poorly differentiated, stage 3c, left ovary	46,XX[93]	rev ish enh(2q31q32,3q25q26,4q13,4q21,4q24q28,5p14,6p, 6q12q23,7q21,7q22q31,8q,9p13p24,10p11,11p14p15, 12p11p13,15q21q23,15q24),dim(Xp11,1p32p36,2p14p15, 2p16p21,2p22,3p21,4p16,5q23q31,7p21p22,9q34, 10q25q26,11q13,11q23,12q13,12q23q24,13q12,13q33q34, 14q13q24,14q31q32,16p11p13,16q21q24,17p,17q,19p,	MSS

			19q13,22q11q13)	
24b/03-519 ***	right ovary	46,XX[66]	rev ish enh(2q22q24,2q31q34,3q13,4q13q32,5p14p15, 5q14q21,6p12p21,6p22,6p24,6q14q21,6q22q23,7q22, 7q31q32,7q33,8q,9p13p24,10p11,10p12p14,11p14p15,12p,1 8q22,20p12p13),dim(Xp11p21,Xp21p22,Xq12q13, 1p31p36,3p21,5q23q31,8p12,8p21,8p22p23,10q21q24, 10q25q26,11q23,12q22q24,16p11p13,16q22q24,17p11p13,1 7q11q25,19p13,19q,22q11q13)	ND
25a/03-1003 ****	serous papillary carcinoma– moderately and poorly differentiated, stage 3c, left ovary	36~44,del(4)(p12),**add(11)(p15)**,add(19)(p13),**add(19)(q13)**,inc[cp20]	rev ish enh(1q21q31,2q22q37,3p,3q12q13,4q12q13,5p,6p24, 7p11p12,7q,8q22qter,9p21p22,9p23,9q32q34,10p11, 10q11q21,11p11p14,11q14,12p,14q22qter,15q24qter, 18q11q12),dim(1p31p32,1p36,2p14p22,2p24pter,2q11q21, 4q24q31,4q32qter,5q12qter,6q16q23,8p,8q11q21,11p15, 11q23qter,12q24,13q21qter,15q13q22,16p,16q13qter,17p, 17q11q23,18q21qter,21q21qter,22q),amp(2q24q32)	MSS
25b/03-1004	right ovary	46,XX[52]	rev ish enh(1p13p21,1p34p35,1q21q32,1q41,2p13,2q22q37, 3p12p21,3p21p25,3q12q22,3q24q28,4p15,5p,6p21,6q12q14, 6q24qter,7q11qter,8q22qter,9p21pter,10q21,10q25qter, 11p11p14,11q14q22,12p,12q14q15,12q21,14q21qter, 15q25qter,18q12),dim(Xq21q26,1p31,2p22pter,2q11q14, 4q25q31,4q33qter,5q21qter,6q21q22,7p21pter,8p,8q11q21, 11p15,11q23qter,12q24,13q21qter,15q11q23,16q22qter,17p, 17q11q24,18q21qter,19p13,19q,21q,22q),amp(2q24q32, 7q35q36,8q23qter)	MSS
25c/03-1005 ****	omentum	39~41,del(1)(p31),der(11) **add(11)(p15)**dup(11) (q14q22),**add(19)(q13)**, ?add(22)(q13),inc[cp9]/ 75~79,idemx2[3]	rev ish enh(1q21q31,2q22q37,3p14p26,5p13p15,6p25, 6q23q26,7q,8q22qter,9p12p21,9p23p24,9q32q33,10q11, 11p12p14,12p11pter,14q22qter,15q24qter,18p11), dim(1p22p31,2q11q21,4p16,4q24q28,4q31q35,5q21qter, 6q21q22,8p,8q11q21,11q23q25,13q21qter,15q15q22,16p, 16q13qter,17p11,17q11q22,18q21q23,21q21q22,22q11q12,	MSS

			22q13),amp(12p13)	
26a/03-1178	mucinous cystadenocarcinoma – well differentiated, stage 2c, left ovary	46,XX[7]	rev ish enh(4p15,7p21),dim(1p32p36,7q11,11q13,16p11p12, 17q11,19)	MSS
26b/03-1179	right ovary	47,XX,+add(1)(p13),add(11)(q13)[5]/ 46,XX[15]	no imbalances	MSS
27a/04-24	endometrioid and serous papillary adenocarcinoma– moderately and poorly differentiated, stage 2c, right ovary	65~79<3n>,X,-X,-X,del(3)(p11),**-4,-5**,**i(8)(q10)**, der(13;14)(q10;q10), add(15)(p11),inc[cp9]	rev ish enh(1p22,1p31,1q23q25,2p13p25,2q21q37,3p12, 3p13,3q12q21,3q22q26,4q12q34,5p,6p21,6q12q22,7p21p22, 7q21q31,8p11,8q,11p11p14,11q14q22,18q11q23), dim(Xp11p21,Xp22,1p34pter,1q42,6q25q27,8p12pter, 9p12p21,9q21qter,13q12q14,13q33q34,14q21qter,17,19p13, 19q,22q11q13),amp(8q13q22,8q22q23)	MSS
27b/04-26	left ovary	58~73<3n>,XXX,**-4,-5**,-5, +8,**i(8)(q10)**x2,-14,-19, inc[cp10]	rev ish enh(1p13p32,1q21q41,2q22q24,2q32q33,3p12p14, 5p14p15,6p12,6q12q24,7p14pter,7q21,7q31q32,8p11,8q,11p, 11q14q23,11q23q25,12p,12q12,12q21,14q11q13,15q21q22, 15q24q26,16q12q21,18q11q12,18q22qter,20p11p13,21q21), dim(X,1p34p36,3q28q29,4q31qter,5q11q14,7q36,8p12pter, 9p12p23,9q21qter,10q,12q24,13q12q21,13q33qter, 14q21qter,17,19p13,19q,22q),amp(8q13qter)	MSS
28a/04-101 ****	clear cell and serous papillary adenocarcinoma, stage 3b, right ovary	63~70,add(12)(p13),add(19)(q13),inc[cp9]	rev ish enh(Xp21p22,Xq13q21,Xq22q24,Xq25q27,1p31,2p, 2q11q14,2q22q24,2q32,2q33,3,4p15,5p13p15,5q11q34, 7p13p14,7p15p21,8p11,8q,9p13p23,10p11p12,10p13p15, 10q11q23,13q22q31,13q31q32,20q,21q21q22),dim(1p32p36, 1q25q43,2q37,6p21p22,7q11,7q21q22,9q22q32,9q33q34, 10q23qter,11p,11q12q13,12q13q14,12q23q24,13q12q13, 14q11q24,15q11q24,16p11p13,16q,17p11pter,17q11q22,18q, 19p13,19q13,20p11p12,22q11),amp(3q24q26,10q21q22)	MSS

28b/04-102	left ovary	67~69,inc[6]	rev ish enh(Xp11p21,Xq21,Xq22q25,Xq26q27,1q23q24, 2p11,2p12p25,2q11q14,2q23q24,3p12,3p13p14,3p21p26,3q, 5p13p15,5q11q23,5q31q34,6p23p25,6q15q16,7p13p21, 7q21q32,8q,9p13p24,10q21q22,20q),dim(1p31p36,1q31q43, 2q37,4q34qter,6q24q26,9q22q32,9q33qter,10p11p12, 10q23qter,11p11p15,11q12q13,12q13,12q23q24,13q12q14, 14q,15q11q24,16p11p13,16q13qter,17p,17q11q23,18q, 19p13,19q13,20p11p13,21q22,22q11q13),amp(10q21q22)	MSS
29a/04-186<br>****	endometrioid adenocarcinoma - poorly differentiated, stage 2c, right ovary	67~73,del(X)(q23),del(1)(q21),?add(11)(p15), der(19)t(1;19)(p32;p13), inc[cp8]	rev ish enh(Xq12,1p12p21,1p31p32,1q,2p12p13,2p23p25, 2q22q24,3q,4p15,4q26q28,5p14,5q14q35,6p11p25,6q12q15, 7q22qter,8q21qter,9p12p13,9q13q21,10p,10q11q22,11q, 12p11p12,12p13,12q12q14,14q11q13,15q23qter,16q12q21, 17q22qter,18p11,18q11q21,20q12qter),dim(Xq21q23, 1p22p31,3p13p24,4p12p14,4q12q25,4q31qter,5q11q12, 6q22q24,7p13p22,7q11,8p12pter,9p21p23,9q22qter, 10q22q24,11p11p15,13q,14q24q31,17p,17q11,19p13,19q, 21q,22q),amp(8q22qter)	MSS
29b/04-187	left ovary	46,XX[3]	rev ish enh(Xp11,Xq12,Xq13,Xq23q27,1p13,1q,2p11p12, 2q13q14,2q22q24,2q36,3p13,3p14,3q,5p13pter,5q32q35, 6p12pter,6q16,7q11,7q32qter,8q22qter,9p12p21,9q13q22, 10p11,10p12pter,11q12q14,15q13qter,16p11p13,16q12q24, 17q22,17q24q25,18p11,18q12q21,19p,19q13,20p11p12, 20q11q13),dim(1p22p31,1p35pter,2p16,3p21p22,4p, 4q12q26,4q31qter,5q11q13,6q22qter,7p14pter,8p12pter, 9q31qter,10q22q24,11p12pter,12q15q24,13q33qter,17p, 17q11,19q13,22q),amp(1q32qter)	MSS
30a/04-919<br>****	serous adenocarcinoma, stage 3c, right ovary	38~42,**add(3)(p24)**,**add(19)(p13)**,**+r**,inc[cp10]	no DNA available	--

30b/04-920	left ovary	39~46,X,del(1)(q21),**add(3)(p24)**,add(4)(q34),**add(19)(p13)**,**+r**,inc[cp9]/74~85, idemx2[cp10]/46,XX[34]	no DNA available	--
31a/04-1049	endometrioid adenocarcinoma - poorly differentiated, stage 4, left ovary	46,XX[8]	rev ish enh(8q11q24),dim(9q34)	MSS
31b/04-1051	right ovary	42,XX[42]	rev ish enh(3p12,3q12q13,5q14,5q21,6p11p12,6q12q21, 6q21q23,9p24,13q21q31,21q21),dim(2q37,3p21,7q35,9q34, 10q25q26,11q13,12p13,12q23q24,14q31,15q22q24, 16p11p13,16q22q24,17p12p13,17q11q21,17q22q25,19p13, 19q13,20q13,22q)	MSS
32a/04-1066	serous papillary adenocarcinoma - moderately differentiated, stage 4, right ovary	culture failure	rev ish enh(1p21p31,1q21q41,2p16,2q21q32,3p12p14,3p24, 3q12q26,4p12p16,4q12q31,5p12p15,5q14q31,6p12,6q12q24, 7q31,8q,11p15,11q14q22,12p11p13,13q14q31,18q11q12), dim(Xp11p21,Xp22,Xq12q21,Xq27q28,1p33pter,2q37, 6q25qter,7q11,7q36,8p21pter,9p12p13,9q21qter,10q26, 11q13,12q23qter,14q32,15q22q23,16,17p,17q11q21,18q22, 19,20q11q12,21q22,22q),amp(8q13q23)	MSS
32b/04-1067	left ovary	culture failure	not informative	ND

* Stage according to FIGO (International Federation of Gynecology and Obstetrics).
**MSS: microsatellite stable, MSI-L: microsatellite unstable-low; ND: not detectable.
*** Tumors from patients who had received neoadjuvant therapy.

****Tumor karyotypes also published in Genes, Chromosomes and Cancer 2009, 48(2):184-93.
